# Finding Community: A Thematic Analysis of Resilience During the Pandemic

**DOI:** 10.13023/jah.0703.02

**Published:** 2025-09-01

**Authors:** Myles Kwitny, Quinn Richards, Grace DiGirolamo, Shannon Freedman, Sophie Wenzel

**Affiliations:** Virginia Tech; Virginia Tech; Virginia Polytechnic Institute and State University

**Keywords:** Appalachia, community health, COVID-19, marginalized populations, resilience, rural health, southwest Virginia

## Abstract

**Introduction:**

The Covid-19 pandemic impacted those within rural Appalachian communities, particularly people with marginalized identities.

**Purpose:**

Through interviewing 44 participants from New River Valley, an Appalachian community in Virginia, this study sought to understand how resilience impacted individuals' experiences during the pandemic.

**Methods:**

Participants belonged to one of the following groups: African American, Hispanic, older adults, or people who use drugs. Participants were required to reside in the New River Valley and be older than 18. Each participant was interviewed either in person, by phone, or over video call. Interviews were recorded, transcribed, then thematically analyzed for themes of resilience. Taguette was used to create tags in the interview transcripts.

**Results:**

Seven themes were identified: pastimes, friends and neighbors, family, employers and employment, faith, community support, and hope. The primary purpose of this analysis was to understand where participants found resilience despite barriers created or perpetuated by the pandemic. The secondary purpose was to apply the themes to address future recommendations and interventions.

**Implications:**

Results of this study inform helpful interventions for these populations including allocation of funding for social supports, development of continued community building, and policy development that supports education and the reduction of health inequities for people of marginalized identities.

## INTRODUCTION

In the United States (U.S.), older adults,^1^ racial minorities,^2^,^3^ and people who used drugs (PWUD)^4^ all saw higher mortality rates than other populations during the pandemic. Disparities in racial mortality rates were further exacerbated in rural areas; African-American and Hispanic populations in rural counties saw higher Covid-19-related mortality rates compared to other populations in the same areas.^5^ There was an overall increase in overdose-related deaths in 2020 for PWUD.^4^ African-Americans who used drugs specifically saw the biggest increase in overdose related death.^4^ One factor that may be protective for individuals of marginalized communities is resilience.

Resilience is a mechanism during which individuals who face challenges develop positive strategies to combat their struggles.^6^ Resilience has been attributed to both physical and environmental factors. Physical factors involve the nervous system, immune responses, and genes; environmental factors can include cultural influences and social factors. Resilience is impacted by resources, support systems, and components of institutional structure.^6^ Social support, faith,^6^,^7^ and positive emotions^6^,^8^ have all contributed to resilience.

Communities of color, especially African-American individuals, faced physical and environmental factors that increased stress both prior to and during the pandemic. Factors such as religion and community connectedness were shown to positively impact resilience in these communities.^9^ Older adults have shown to have strong resilience through coping mechanisms, such as social support, daily routine, and staying occupied through activities.^8^

This article explores themes of resilience during the COVID-19 pandemic across people identifying with one or more of these populations: African-Americans, Hispanics, older adults, and PWUDs. The primary goal is to describe how individuals in Virginia’s (VA) New River Valley (NRV), a rural Appalachian region, found resilience despite structural, social, and physical barriers which were created or perpetuated by the pandemic. The second goal was to apply these themes of resilience to future recommendations and interventions for the NRV.

## METHODS

The author team sought to understand challenges faced by those living with marginalized identities in the pandemic and what adaptations could be made to combat negative experiences. The participants included those belonging to one or more of these four groups: African-Americans (n=9), Hispanics (n=12), older adults (n=11) and PWUD (n=12). Note that although these participants were recruited and identified as being a member of one of these four groups, these identities are not necessarily exclusive. The authors acknowledge participants’ intersectionality, and even though they are categorized as belonging to one of these four groups to ensure that these groups’ voices were represented in this research, participants may have identified with more than one of these groups. Participant eligibility included being over 18 years old, living within the NRV, and identifying as a member of one of the four populations. Project participants were locally recruited through various organizations, such as health departments, churches, and non-profit organizations within the NRV. Accessibility was increased by utilizing both virtual and on-site recruitment.

Participants were interviewed in-person, over the phone, or on the online Zoom platform in the Spring of 2021 by members of the research team. Interviews were conducted in both English and Spanish. Participants were compensated with a $25 gift card. Additionally, each participant chose a pseudonym for privacy. Interviews included optional demographic questions (Table 1).

Interview questions revolved around community impacts observed during the pandemic, impacts on physical and mental health, changes in daily life, impacts on household, important people in their lives, community resources available and needed, and advice for local government.

After transcribing recorded interviews, the study team conducted a thematic analysis looking at themes of resilience. The author group chose to focus on resilience, because when reading the transcripts, themes of resilience persisted throughout all four groups and inspired a deeper exploration of the topic. A hybrid of deductive and inductive coding was used. A series of potential codes was developed ahead of the coding process based on a preliminary review of the transcripts and after reading literature on resilience. New codes that emerged during the data analysis process were compiled and summarized. Coding was conducted using Taguette. Each transcript was assigned three coders: two primary coders, who separately coded, and a tertiary coder, who ensured concurrence between the two primary coded transcripts. After coding was finalized, a thematic analysis process was conducted using Braun and Clarke’s^10^ method. This thematic analysis resulted in seven themes focused on resilience. Quotes in Spanish were translated to English.

### Ethics Approval

This study was approved by the Virginia Tech Institutional Review Board (#20-1002).

## RESULTS

Participants’ mean age was 47 years (range 20–90), with 57% women and 41% men. Participants self-reported race and included 16% African-American, 2% Indigenous, 45% white, 7% more than one race, and 30% “not reported”. Thirty-four percent identified as Hispanic and 45% as non-Hispanic. Household income ranged greatly, with 30% reporting $0–$10,000 (Table 1).

Through the thematic analysis, seven common themes were identified as they related to resilience during and after the COVID-19 pandemic: pastimes, friends and neighbors, family, employers and employment, faith, community support, and hope. The thematic analysis was conducted across the four groups to show that themes of resilience span boundaries beyond race, age and other lived experiences.

### Pastimes

Social distancing and quarantine policies kept many people at home during the pandemic. While secluded at home, participants picked up new pastimes or revived old ones. These activities contributed to their resilience and their mental health. Many people turned to physical activities including walking, yoga, and calisthenics, which were often conducted outside. Other outdoor activities such as gardening, birding, and sitting outside had positive impacts. One participant, Frankey, enjoyed gardening. He shared this statement when asked about his newfound hobbies:

“I have grow boxes and I put em’ on the deck and I grow my tomatoes and cucumbers and beans and peppers and that’s helpful to me. Gives me something to look forward to...,”

Participants similarly enjoyed indoor activities such as writing, crafts, and puzzles. Reading and learning new skills allowed participants to keep their minds engaged and to educate themselves. Games and puzzles were a way to connect with others virtually or in-home during quarantine. Andrea connected with her daughter through letter writing:

“The cool thing is, is me and my daughter started writing letters to each other... like we do little notes to each other. Fold them like in high school. Throw them under each other's door.”

Additionally, having time alone, especially during quarantine, allowed participants to reflect on their values. Many found this isolation to strengthen their mindfulness skills and reflect on what they were thankful for. Self-reflecting helped with participants’ mental health and allowed them connection to the world around them. Blair was “thinking and writing about the sense of place in relationships to nature.”

### Friends and Neighbors

For many participants, their relationships with people around them changed significantly. Connections between friends grew stronger. For many, simply having another person to speak to, even though digital means, helped combat loneliness. Maxwell found support in friends:

“A lot of support came from friends, right. People who are also just holding wonder and curiosity about life meaning, spirituality, whatever. So it didn’t come from organized religion or particular spaces. It came from just authentic connection in people who see me loving and can hold me.”

The changing climate gave friends an opportunity to understand each other and empathize in ways they had not before. Individuals in recovery from substance abuse leaned on each other for support to ensure relapses were less likely. Friends helped participants connect with others outside their social circles to understand their own roles in the community.

Neighbors were an excellent source of strength. Their proximity provided a sense of safety and camaraderie. From watching each other's homes to spending quality time together, many participants expressed gratitude towards neighbors. Annie found herself helping one of her older neighbors. She described, “we’ve [she and the neighbor] helped each other... I help her sometimes and she’s got this little dog and I had to walk her dog outside for her.”

### Family

Family members provided multiple different types of assistance to participants including financial, emotional, and task-based support. Tasks performed by family members included grocery shopping, chores, and transportation. This support allowed participants to feel more secure during the pandemic. The emotional support provided by family members allowed participants to continue to stay resilient and work through challenges. Stephanie found support in her family during her time in recovery:

...my family has always been a support to me even, you know, during my active use. ... they never gave up on me and they never disowned me.... My mom is my rock. She supports me no matter what I do and tries to help me to the best of her ability. She didn’t live the life of an addict, but she watched her daughter go through it and that was enough.

Quarantine allowed families who cohabitated to spend long periods of time together. They engaged in activities together and independently. They reflected on how being together for extended periods of time required them to develop strong communication skills. This was essential to respect each other's space but also to check in on each other's emotional and mental well-being. Family members who were in different households began to meet virtually over video calls or speak over the phone more often. This helped build relationships in positive ways.

### Employers and Employment

The pandemic impacted employment of individuals including how they worked, workplace safety, and fluctuation in pay and hours. Participants held a variety of jobs and employment types. Some transitioned to working remotely while others continued to work in-person. While employment varied, many participants found resilience through their new work settings and support from employers and fellow employees.

Many participants discussed employers adjusting schedules and workplaces so workers would remain employed. Some employers continued providing benefits and 40 hours of pay even when there was limited work. Having consistent benefits and paychecks allowed participants to continue to support themselves and their families. For example, Mario reflected on his employer's support:

“They were supporting us because even though there was nothing, I mean we had practically no sales...They supported us with hours and supported all of us. In other words, they didn’t leave anyone with nothing.”

Many participants who continued to work in-person also described adjustments their employers made to ensure employee safety. Participants described employers taking temperatures, requiring masks, and sending employees home if they were showing symptoms of COVID-19.

For those who worked from home, many described saving money because they were not commuting to work or spending money outside of the house. Many employers provided resources for individuals to transition from working on-site to working remotely. Employers allowed participants to have flexibility in their work schedule and more time off to prevent burnout. Working from home allowed flexibility to do other tasks, such as cooking and laundry, that often would be completed after work hours or over weekends. Noemi found positives in having this flexibility:

“The world slowed down. I had increased flexibility, and I was able to enjoy my home because even though I've been living there for years, I was out of it more than I was in it.”

### Faith

Faith played a role in helping participants remain resilient. Believing in a higher power gave many participants hope and allowed them an outlet with which to express their frustrations or their desires for the future.

Flor, reflecting on her spirituality, shared that “He [God] has given me strength...God never left me alone, God was always there giving me strength, helping me.”

Individuals who attended organized services found socialization almost as crucial as their relationship with their spirituality. Having specified roles in the organization gave some participants a feeling of well-being and provided human connection. Being amongst like-minded people or having a reminder that they were not alone went a long way. Will Smith described support from his Jewish community:

“The Jewish community always looks out for its own. That is one thing that I think is probably well-documented and most people are aware of. And that was when I started getting a lot of the support that I needed for different things was when I reached out specifically to that community.”

Church leadership served as emotional support for those who missed attending services in-person. Maria Austin found that “if something’s wrong too, I write my pastor or call him and he helps me, he prays.” Many faith-based groups offered online services or meetings. These allowed socialization and group camaraderie while keeping safety a top priority. Randall Wells "did communion online...we [he and his wife] just have our wine and our bread, and we would just do the service together.”

Another benefit of religious organizations was their ability to provide aid for community members. Members were able to collect resources from physical locations even as services remained virtual. Karina described, “the church gave us a lot of support... if we [she and her family] didn’t have rent or electricity, then we had help from the church.”

Many religious organizations extended their resources to anyone who needed it and helped provide food or other necessities for those who were in need.

### Community Support

Participants turned to the community for support. Often this came in the form of virtual community spaces where participants could connect with like-minded people. For individuals in recovery from substance use, recovery homes and meetings were places of community support that helped with resilience. Some PWUD, specifically, struggled to maintain their sobriety and leaned on their support system. Andrea found online meetings allowed for those in recovery to “hear other people’s stories from all around the world. So it was nice to feel like we weren’t alone and we could talk about things.”

For others, seeking outside help for mental health or advice for coping with general uncertainty was preferable. Participants spoke highly of therapy as a coping mechanism. Maxwell shared that he “started therapy for the first time. And so speaking with the counselor, was super necessary for me... I need some support.”

When participants did leave their homes and go out in the community, often for groceries and other essentials, they felt supported by businesses and people who followed COVID-19 health regulations. Participants noted that they felt connected to the community through talking with workers at stores. Having these conversations helped participants socialize and contributed to feelings of resilience.

Communities came together to provide food, supplies, or conversation to those who needed it. Strong connections flourished, allowing people to support each other on a large scale. Areas that did not have as many resources made efforts to adapt so all members of the community would have what they needed. Damion described “everybody started coming together and really started taking care of each other.”

Participants mentioned activism such as the Black Lives Matter (BLM) movement as areas of community growth and resilience. Justin McMiller, who was in Washington, DC during BLM demonstrations, described his experience seeing people after a demonstration:

“There were people of my generation, so it was like ‘okay, well at least our future is gonna be good, we just gotta make it there’....it was really powerful to see that the strength of it was just like this resilience.”

Many participants described unity in their communities, whether this was geographic areas such as their towns or cities, or virtual/support community spaces. Having a feeling of community and community strength helped participants feel they could stay resilient. Lacy saw this theme in the African-American community.

“You know we, as African Americans have always been able to strive and get past any situation that had been placed on us in this nation. And this (COVID-19) is just another obstacle or hurdle that we have to overcome collectively as a unit and make sure that the loss of life that was felt within our community… they're never forgotten.”

### Hope

The pandemic forced individuals to reimagine what the future would look like in just a matter of weeks or months. Many participants expressed their desire for the return to normal, while others embraced differences the pandemic introduced. As people looked forward, they counted their blessings, considering lockdown and the pandemic as an opportunity to foster personal growth. Living in a recovery community, after being housing insecure, Ella found herself “appreciating my little roof over my head and like my little bed.”

Many participants mentioned their desire to help others. After seeing work accomplished in their communities and through media, or even after receiving help themselves, people hoped to apply their own skills and resources to others and the community. Justin McMiller wanted to

“… continue living life without fear and not restrain myself and doing other goals that I have set for myself and just like prospering and being able to be in a position where I can help other people.”

On a larger scale, participants hoped they could rely on the rest of the world to do their part to end the pandemic. Doug hoped people would “mitigate, wear our masks, social distance, educate each other and be there to support each other. I think we can get through this.”

Others longed for a shift in society regarding how people treat each other and how they identify with their community or the world at large. Thomas hoped that “either we slow down somewhat, lower expectations, or have more understanding, and then work together towards a new normal because there’s no going back to what is was.”

## DISCUSSION

The goal of this project was initially to learn about marginalized peoples’ experiences during the pandemic and to think through interventions to address disparities in future public health crises, specifically in Appalachia. A topic that emerged from the interviews was the idea of resilient people and communities, which has been further explored through this study. According to the *Resilient Appalachia* project,^11^ Appalachian people and communities have a long history of resilience in the face of social, financial, environmental and other hardships. Participants in this project found many ways to be resilient in the face of a global pandemic. Participants across all the groups described a range of resiliency strategies categorized into seven themes: pastimes, friends and neighbors, family, employers and employment, faith, community support and hope. These findings show the breadth of resiliency strategies and show that despite differences in identity, race, age and lived experiences, many participants relied on similar sources of strength.

Resilience was seen in both individual activities such as gardening and increased spirituality and collective activities such as connecting with neighbors, friends and family. Participants expressed gratitude for flexible work arrangements, showing the importance of employers in ensuring economic wellbeing and health and safety during public health crises. Participants also mentioned resilient communities, such as those observed during the BLM protests and the importance of recovery communities for PWUD and those in recovery.

These findings build on existing research on resiliency and contribute new rural-specific insights. This study affirms previous findings about the protective effects of social supports, hope, and faith, among other things.^6^,^7^,^8^

This study also offers practical implications and relevant recommendations. Multiple participants mentioned the importance of building a sense of community connectedness, expanding health services, and planning for future pandemics. Participants emphasized having programs that address stereotypes, biases, and health disparities faced by people with marginalized identities. Social, community, and health services that support marginalized communities should continue to be funded and expanded in the NRV. These services should be culturally competent, and providers should be trained in topics including substance use and recovery. There should be bilingual staff who are fluent in Spanish.

Community connection plays an important role in resilience. In the NRV, community spaces such as churches, recovery homes, and schools were important gathering places for marginalized communities. Community spaces can be utilized to distribute health information, build partnerships, and offer health services. They should be accessible by public transportation. Building relationships with leaders in these spaces can help promote and expand accessibility.

Finally, health messaging should be accessible to everyone. This includes distributing messaging in a variety of formats (online, radio, paper, etc.) and multiple languages. Health crisis plans should include funding allocations, health messaging dissemination, community resource lists, community spaces for providing services, and roles of health workers working with vulnerable populations. These plans should be multi-organizational, community-approved, and flexible. Policies that expand public transportation, support employment security, address minority health inequities, and promote affordable and accessible mental health services would be beneficial.

### Limitations

Participants were recruited using a convenience sample or snowball sampling methods, potentially revealing a self-selection bias. This study is context specific to the NRV; however, the participants may not adequately represent the greater population of the region. The small sample size of 44 individuals limits generalizability. Finally, retrospective reflection could lead to recall bias.

## CONCLUSION

Future studies could include quantitative components or further explore how resiliency may differ for intersectional identities not explored here. Future studies could also look at long-term impacts of community-based resilience on mental health, substance use and health equity.

Despite structural challenges faced during the pandemic, participants across marginalized communities demonstrated resilience through interpersonal connections and activities and intra-personally. These findings offer insight into how rural marginalized populations adapt to crises and may inform future emergency preparedness activities and community resiliency initiatives. The authors hope this study will help promote health programming and policy that supports marginalized individuals living in the NRV and other Appalachian regions.

**SUMMARY BOX**

**What is already known about this topic?**

Resilience is a mechanism during which individuals who face challenges develop positive strategies to combat their struggles. Resilience is impacted by resources, support systems, and components of institutional structure. Social support, faith, and positive emotions have all contributed to resilience.

**What is added by this report?**

This article explores themes of resilience during the COVID-19 pandemic across people with one or more marginalized identities. Through the thematic analysis, seven common themes were identified as they related to resilience during and after the COVID-19 pandemic: pastimes, friends and neighbors, family, employers and employment, faith, community support, and hope.

**What are the implications for future research?**

Despite structural challenges faced during the pandemic, participants across marginalized communities demonstrated resilience through interpersonal connections and activities and intra-personally. These findings offer insight into how rural marginalized populations adapt to crises and may inform future emergency preparedness activities and community resiliency initiatives. The authors hope this study will help promote health programming and policy that supports marginalized individuals living in the NRV and other Appalachian regions.

## Figures and Tables

**Table 1:** Demographics of Participants

*Mean Age*	47 (age range: 20 – 90)
**Demographic**	**Total (%)**
**Gender**	
Women	25 (57%)
Men	18 (41%)
Not reported	1 (2%)
Total	44
**Race**	
African-American	7 (16%)
Indigenous	1 (2%)
White	20 (45%)
More than one race	3 (7%)
Not reported	13 (30%)
Total	44
**Ethnicity**	
Hispanic	15 (34%)
Non-Hispanic	20 (45%)
Not reported	9 (20%)
Total	44
**Household Income ($)**	
0–10,000	13 (30%)
10,000–25,000	9 (20%)
25,000–50,000	7 (16%)
50,000–100,000	2 (5%)
100,000 or above	5 (11%)
Not reported/Prefer not to answer	8 (18%)
Total	44
**Highest Level of Education**	
No high school diploma	5 (11%)
High school graduate/GED	15 (34%)
Trade school	2 (5%)
Bachelor’s degree	5 (11%)
Bachelor’s degree and higher	15 (34%)
Not reported	3 (7%)
Total	44
